# Resonant X-ray emission spectroscopy using self-seeded hard X-ray pulses at PAL-XFEL

**DOI:** 10.1107/S1600577523007312

**Published:** 2023-09-22

**Authors:** Tae-Kyu Choi, Jaeku Park, Gyujin Kim, Hoyoung Jang, Sang-Youn Park, Jang Hyeob Sohn, Byoung Ick Cho, Hyunjung Kim, Kyung Sook Kim, Inhyuk Nam, Sae Hwan Chun

**Affiliations:** a XFEL Division, Pohang Accelerator Laboratory, POSTECH, Pohang, Gyeongbuk 37673, Republic of Korea; b Photon Science Center, POSTECH, Pohang, Gyeongbuk 37673, Republic of Korea; c Department of Physics and Photon Science, Gwangju Institute of Science and Technology, Gwangju 61005, Republic of Korea; d Department of Physics, Sogang University, Seoul 04107, Republic of Korea; ESRF – The European Synchrotron, France

**Keywords:** X-ray free-electron laser (XFEL), self-seeding, resonant X-ray emission spectroscopy (RXES), von Hamos spectrometer

## Abstract

Self-seeded hard X-ray pulses at PAL-XFEL were used to commission a resonant X-ray emission spectroscopy experiment with a von Hamos spectrometer. The self-seeded beam, generated through forward Bragg diffraction of the [202] peak in a 100 µm-thick diamond crystal, exhibited an average bandwidth of 0.54 eV at 11.223 keV. A coordinated scanning scheme of electron bunch energy, diamond crystal angle and silicon monochromator allowed us to map the Ir *L*β_2_ X-ray emission lines of IrO_2_ powder across the Ir *L*_3_-absorption edge, from 11.212 to 11.242 keV with an energy step of 0.3 eV. This work provides a reference for hard X-ray emission spectroscopy experiments utilizing self-seeded pulses with a narrow bandwidth, eventually applicable for pump–probe studies in solid-state and diluted systems.

## Abstract 2

Resonant X-ray emission spectroscopy using self-seeded hard X-ray pulses with precise photon energy control was successfully demonstrated at the PAL-XFEL. This achievement proves the feasibility of a self-seeded beam for advanced X-ray spectroscopy experiments.

## 1. Introduction

X-ray free-electron lasers (XFELs) produce X-ray pulses with high brightness, long coherence length and ultrashort pulse duration (Emma *et al.*, 2010 ▸; Ishikawa *et al.*, 2012 ▸; Kang *et al.*, 2017 ▸; Prat *et al.*, 2020 ▸; Decking *et al.*, 2020 ▸). These features surpass the intrinsic limit of synchrotron light sources, enabling advanced X-ray measurement techniques such as femtosecond time-resolved X-ray scattering and spectroscopy, coherent X-ray imaging and serial femtosecond crystallography (Chapman *et al.*, 2006 ▸; Seibert *et al.*, 2011 ▸; Zhang *et al.*, 2014 ▸; Kim *et al.*, 2015 ▸; Gruhl *et al.*, 2023 ▸). The most commonly used method to generate femtosecond X-ray pulses is through the self-amplified spontaneous emission (SASE) process starting from the electron beam shot noise (Huang & Kim, 2007 ▸). In this process, the electron beam, accelerated close to the speed of light, interacts back and forth with X-rays emitted in undulators, and forms micro-bunches that are spaced apart by the X-ray wavelength (Margaritondo & Ribic, 2011 ▸). These micro-bunches radiate the X-ray photons in phase, resulting in an XFEL pulse with significantly amplified intensity and nearly full transverse coherence (Gutt *et al.*, 2012 ▸; Yun *et al.*, 2019 ▸). However, the stochastic XFEL radiation originating from the shot noise leads to large intensity fluctuation and poor longitudinal coherence (or temporal coherence). For instance, a SASE spectrum contains tens of spikes corresponding to multiple temporal modes, with their intensity randomly varying for subsequent X-ray pulses (Lee *et al.*, 2015 ▸; Kujala *et al.*, 2020 ▸). Although a double-crystal monochromator (DCM) can filter out the temporal mode in the hard X-ray regime, the expense of photon flux using a high index of Bragg reflections for the monochromator crystals restricts XFEL experiments that require high signal-to-noise ratio and temporal coherence.

The self-seeding technique is a promising method for narrowing the XFEL bandwidth while keeping high photon flux in the hard X-ray regime. This process involves a monochromator crystal in either Bragg reflection (Saldin *et al.*, 2001 ▸; Inoue *et al.*, 2019 ▸) or forward Bragg diffraction (Geloni *et al.*, 2011 ▸; Amann *et al.*, 2012 ▸) to filter a seed from the SASE pulse generated in upstream undulators. The seed pulse is then amplified in downstream undulators by the electron bunch that is temporally overlapped with it. The Pohang Accelerator Laboratory X-ray Free-Electron Laser (PAL-XFEL) has successfully delivered its self-seeded beam using a diamond monochromator crystal, over a wide photon energy range of 3.5 to 14.6 keV (Nam *et al.*, 2021 ▸; Min *et al.*, 2019 ▸). The self-seeded beam produced at the PAL-XFEL exhibits high peak brightness, narrow bandwidth and excellent stability, opening up new research opportunities in X-ray science (Eom *et al.*, 2022 ▸).

This report presents the first commissioning results of a resonant X-ray emission spectroscopy (RXES) experiment using the self-seeded hard X-ray beam at the PAL-XFEL. A von Hamos spectrometer was introduced into the X-ray Scattering and Spectroscopy (XSS) hutch, and was used to measure the Ir *L*β_2_ (4*d* → 2*p*_3/2_) fluorescence from IrO_2_ powder, a widely used oxygen evolution reaction catalyst with a 5*d* element (Ping *et al.*, 2015 ▸), whose *L*_3_-absorption edge (2*p*_3/2_ → 5*d*) lies in the hard X-ray regime. We mapped the Ir *L*β_2_ X-ray emission spectrum across the Ir *L*_3_-edge (∼11.22 keV) using a coordinated scanning scheme of the electron bunch energy, the self-seeding diamond crystal angle and the DCM energy. This intensity map of the Ir *L*β_2_ RXES, or 2*p*_3/2_4*d* resonant inelastic X-ray scattering (RIXS), was obtained within an hour at a repetition pulse rate of 60 Hz by scanning the incident photon energy with an energy step of 0.3 eV over a range of 30 eV. We observe spectral features from the 4*d*_5/2_ and the 4*d*_3/2_ electronic states, and also extract a high-energy-resolution fluorescence-detected (HERFD) X-ray absorption near-edge structure (XANES) spectrum from the map. Importantly, the X-ray emission intensity shows a clear correlation with the incident flux in a single-shot pulse, demonstrating the feasibility of time-resolved RXES and HERFD-XANES experiments via the self-seeded hard X-ray beam for the first time.

## 2. Experimental setup at the XSS hutch

**Figure 1:**
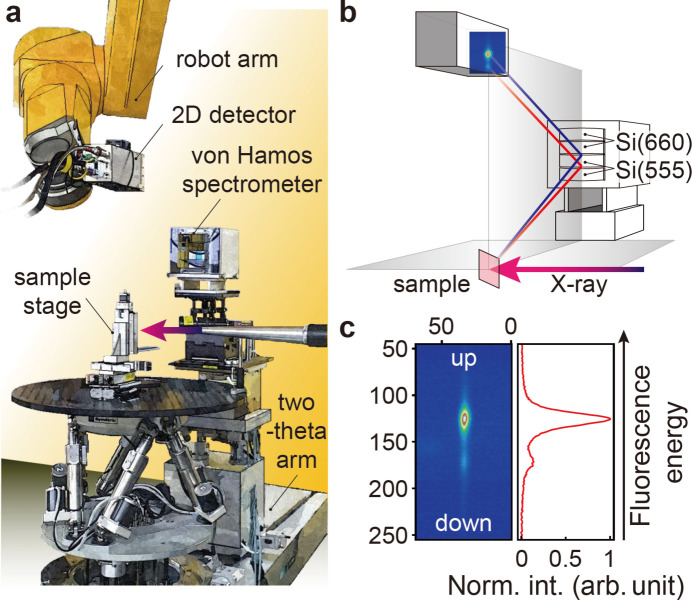
(*a*) Layout of the XES setup at the XSS hutch of PAL-XFEL. A single-column (4 × 1) von Hamos spectrometer is placed on the horizontal two-theta arm of the two-circle diffractometer with its two-theta angle set to 90° to minimize the elastic background signal from the sample. A JUNGFRAU detector is held on the robot arm that is located above the sample stage. (*b*) An illustration of the experimental scheme for the von Hamos spectrometer equipped with two Si(660) and two Si(555) analyzer crystals (radius of curvature = 500 mm). The spectrometer and the detector are positioned on the Rowland circle in the vertical diffraction plane. The emitted X-ray photons are dispersed vertically with higher (lower) energy diffracted at a lower (higher) Bragg angle. (*c*) An averaged 2D detector image of the Ir *L*β_2_ (4*d* → 2*p*_3/2_) fluorescence of the IrO_2_ powder sample, which is dispersed vertically and focused horizontally by one analyzer crystal (left panel). The numbers in the left panel denote pixel numbers. The projection of the image onto the vertical line corresponds to the X-ray emission spectrum normalized with the maximum intensity. The fluorescence energy increases upward (right panel).

The XSS hutch at the hard X-ray beamline of the PAL-XFEL is dedicated to time-resolved X-ray scattering and spectroscopy experiments with the incident photon energy between 2.2 and 15 keV (Park *et al.*, 2016 ▸). The hutch is equipped with a two-circle and a four-circle diffractometer, one of which being selected based on the experimental configuration. The former can accommodate additional equipment on its rotational stages for a specific experimental design, while the latter is primarily used for X-ray diffraction experiments implementing various X-ray reflection geometries for single-crystalline samples. We have built an X-ray emission spectroscopy (XES) setup with the two-circle diffractometer by locating a single-column (4 × 1) von Hamos spectrometer on the horizontal two-theta arm, as illustrated in Fig. 1 ▸(*a*). According to a target fluorescence energy and selected analyzer crystal planes, a two-dimensional (2D) detector on a robot arm is positioned on the Rowland circle in a vertical diffraction plane, and collects the X-ray emission spectrum dispersed by the analyzer crystals on the von Hamos spectrometer (Galler *et al.*, 2019 ▸; Khakhulin *et al.*, 2020 ▸; Sahle *et al.*, 2023 ▸).

For the commissioning experiment, we used IrO_2_ powder and the incident X-ray photon energy chosen across the Ir *L*_3_-absorption edge (∼11.22 keV). The von Hamos spectrometer was equipped with two Si(660) and two Si(555) cylindrically bent crystal analyzers (radius of curvature = 500 mm), as shown in Fig. 1 ▸(*b*). These analyzers were aligned to diffract the Ir *L*β_2_ fluorescence around 10.924 keV at Bragg angles of 62.5° and 64.8°, respectively. The X-ray emission spectra from four analyzer crystals were recorded simultaneously, thanks to a sufficiently large active area of the JUNGFRAU detector (1024 × 512 pixels, pixel size = 75 µm × 75 µm) (Jungmann-Smith *et al.*, 2014 ▸; Redford *et al.*, 2018 ▸; Biednov *et al.*, 2023 ▸). To minimize background elastic scattering signals, we set the two-theta angle to 90°, placed a helium-flowing bag along the X-ray flight paths, and shielded the detector’s active area with lead tape, except for an opening to the spectrometer (Bergmann & Cramer, 1998 ▸; Hayashi *et al.*, 2004 ▸). The left panel of Fig. 1 ▸(*c*) displays an averaged 2D detector image of the Ir *L*β_2_ fluorescence diffracted by one of the Si(660) analyzer crystals. The background-subtracted signal projected onto the vertical line corresponds to the Ir *L*β_2_ X-ray emission spectrum, with its fluorescence energy increasing upward [right-hand panel of Fig. 1 ▸(*c*)].

The energy resolution of the von Hamos spectrometer (Δ*E*_VHS_) is determined by three contributions: Δ*E*_D_ and Δ*E*_cr_ are the Darwin width and stress-induced intrinsic broadening from the cylindrically bent crystal, respectively, while Δ*E*_G_ is the angular divergence associated with the finite vertical size of the detector pixel (*V*_pxl_) and the incident X-ray beam at the sample position (*V*_beam_). These are convoluted assuming each of them is in a Gaussian profile for simplicity (Hayakawa *et al.*, 1999 ▸),

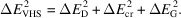

The angular divergence Δ*E*_G_ is estimated using the equation Δ*E*_G_ = *E*_B_Δθ_G_cotθ_B_, where *E*_B_ is the photon energy for the Bragg diffraction at its angle *θ*_B_ on the analyzer crystal, and Δ*θ*_G_ is the angular deviation within the *V*_pxl_ and *V*_beam_. Δθ_G_ is approximated as: 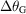 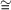 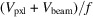 , where *f* is the X-ray flight distance from the sample to the detector. For example, at *E*_B_ = 11.223 keV, the X-ray beam is focused by a set of beryllium compound refractive lenses, resulting in a *V*_beam_ of 30 µm full width at half-maximum (FWHM) at the sample position, while the JUNGFRAU detector has a *V*_pxl_ of 75 µm. For the Si(660) Bragg diffraction at θ_B_ = 59.7°, *f* and Δθ_G_ are 1158 mm and 9.07 × 10^−5^ rad, respectively. Δ*E*_G_ is estimated to be 0.59 eV and is dominant for Δ*E*_VHS_ (Alonso-Mori *et al.*, 2012 ▸).

## 3. XFEL beam transport and diagnostics

**Figure 2:**
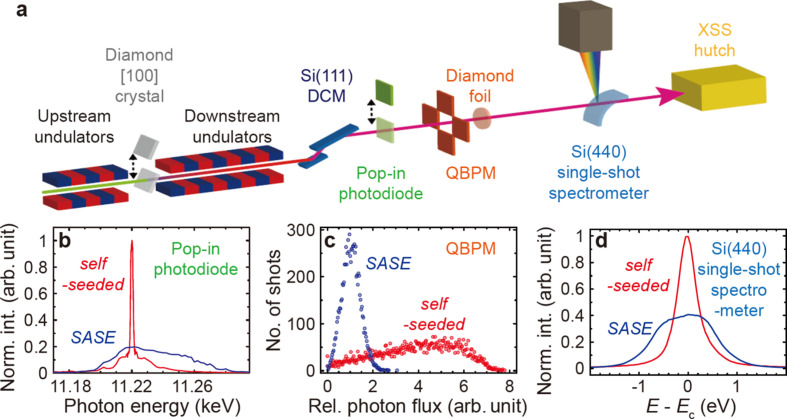
(*a*) A simplified overview of the hard X-ray beam transport and diagnostics at the PAL-XFEL. Self-seeded pulses are generated via the [202] peak of a diamond [100] crystal after the SASE spectrum is optimized to a target photon energy. (*b*) An averaged SASE and self-seeded X-ray spectrum measured with a pop-in photodiode by scanning the Si(111) DCM angle. The target photon energy is 11.223 keV. (*c*) The photon flux distribution of the SASE and the self-seeded pulses at 11.223 keV measured with a QBPM over 9000 shots. The average photon flux of the SASE beam is set to 1. (*d*) The averaged spectrum of the SASE and the self-seeded beam over 3600 shots measured with a Si(440) single-shot spectrometer (*E*_c_ = 11.223 keV). Both spectra are normalized by their area under the curve.

Self-seeded hard X-ray pulses were generated with an electron bunch charge of 180 pC and an undulator *K* parameter of 1.87 with a period of 28 mm (Nam *et al.*, 2021 ▸). A moderate tuning of the self-seeding condition was found to be sufficient for our experiment. A narrowband seed pulse was filtered after eight 5 m-long undulators by forward Bragg diffraction of the [202] peak through a 100 µm-thick diamond [100] crystal (Min *et al.*, 2019 ▸). The seed pulse was then directed by a bandpass filter with a delay of tens of femtoseconds, and overlapped in time with the detoured electron bunch by a magnetic chicane to amplify the narrow seed spectrum (Lee *et al.*, 2015 ▸). The incident XFEL beam was assessed by diagnostic tools placed along the beamline from upstream to downstream as depicted in Fig. 2 ▸(*a*). The spectral intensity profile after the downstream undulators was checked using a pop-in photodiode as a function of incident photon energy deduced from the Bragg diffraction angle of Si(111) DCM crystals. The self-seeded beam averaged over 60 shots showed a sharp peak intensity at 11.223 keV on top of a broad background, which is around five times higher than that of the SASE beam [Fig. 2 ▸(*b*)]. The energy resolution of this measurement is determined by the Darwin width of the Si(111) DCM, which is ∼1.5 eV at 11.223 keV (Als-Nielsen & McMorrow, 2011 ▸). A precise assessment of the narrower bandwidth of the self-seeded beam can be achieved using a single-shot spectrometer with higher energy resolution.

When the DCM crystals are adjusted to the angle that maximizes the peak intensity, the photon energies outside the Darwin width of the Si(111) DCM are removed from the self-seeded beam. The incident photon flux filtered by the DCM was recorded using photodiodes in a quadrant beam-position monitor (QBPM), which detect backscattered X-ray signals from a 2 µm-thick diamond foil and provide the most reliable measurement of the incident photon flux at the hard X-ray beamline of PAL-XFEL (Alkire *et al.*, 2000 ▸; Tono *et al.*, 2011 ▸). Figure 2 ▸(*c*) shows the photon flux distribution of the SASE and the self-seeded X-ray pulses over 9000 shots at 11.223 keV. Over 80% of the self-seeded X-ray pulses had higher flux than the SASE X-ray pulses, and the average photon flux was relatively about five times higher than that of the SASE X-ray pulses, which is consistent with Fig. 2 ▸(*b*).

The incident X-ray spectrum was monitored with a single-shot spectrometer further downstream of the DCM (Zhu *et al.*, 2012 ▸; Kujala *et al.*, 2020 ▸). This spectrometer employs a Si(440) curved crystal (radius of curvature = 100 mm), and an Andor detector (ZYLA5.5X-FO, 2560 × 2160 pixels, pixel size = 6.5 µm × 6.5 µm) resolves the energy-dispersed spectrum in ∼0.05 eV for the photon energy centered (*E*_c_) at 11.223 keV (Kim *et al.*, 2022 ▸). Figure 2 ▸(*d*) compares the area-normalized spectra of the SASE and the self-seeded beams that are averaged over 3600 shots. The spectral profile of the self-seeded beam after the DCM at the peak energy consists of the self-seeded portion and the SASE background, both filtered through the Darwin width. However, the self-seeded portion outweighs the SASE background, leading to the spectrum in Fig. 2 ▸(*d*) displaying minimal SASE background and instead revealing the narrower self-seeded beam. We found that the average bandwidth of the self-seeded beam was 0.54 eV by fitting a Gaussian profile, about three times narrower than the Darwin width of the Si(111) DCM crystals, which limits the monochromated SASE beam bandwidth to ∼1.5 eV. The incident pulse energy of the self-seeded beam after the DCM was recorded by another QBPM located ∼3.3 m upstream of the sample position. The average pulse energy was 355 µJ during the experiment and the air path from the QBPM to the sample position was shorter than 0.3 m.

## 4. Photon energy control system for self-seeded hard X-ray beam

**Figure 3:**
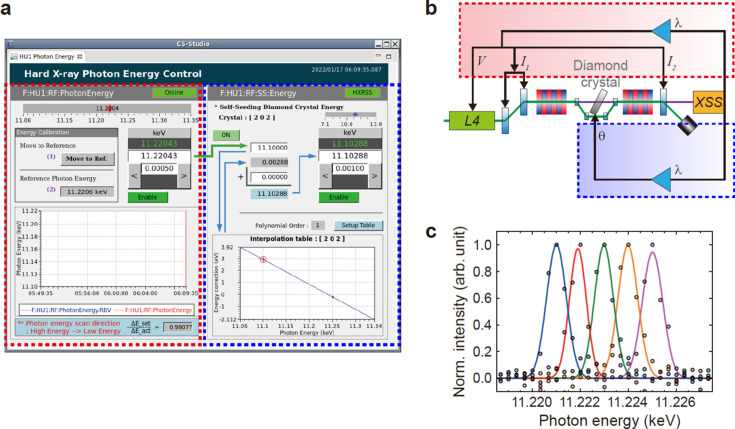
(*a*) The hard X-ray photon energy control system at the PAL-XFEL and (*b*) a schematic illustration of its operation. The optimization and interpolation of the diamond crystal angle were accomplished within the range Δ*E*/*E* ≃ 2% centered at 11.220 keV. Given the target photon energy λ, the red dashed box changes the electron bunch energy accordingly (*V*, *I*_1_, *I*_2_) and the calculated diamond crystal angle θ is compensated from the calibration table in the blue dashed box. The green line indicates the electron beam path and the purple line shows the self-seeded beam path to the XSS hutch. (*c*) The system was demonstrated with the von Hamos spectrometer by recording elastic scattering signals from 11.221 to 11.225 keV with an energy step of 1 eV. Each signal is fitted with a Gaussian curve.

The photon energy control system for the self-seeded hard X-ray beam at the PAL-XFEL is designed to maintain an average photon flux while scanning the incident photon energy over a range of ±1%. This system has two main functions as shown in Fig. 3 ▸(*a*). First, it optimizes the electron bunch energy for the broad SASE background to be centered at the photon energy (left panel). Second, it tunes the diamond [100] crystal angle to maximize the sharp peak intensity at the center of the spectral background (right panel). Once the electron bunch energy and the diamond crystal angle are optimized for selected photon energies within the range of interest, the photon energy control system uses the interpolated self-seeding parameters to maintain the average photon flux stable with an energy precision of ∼0.1 eV around 11.223 keV (Δ*E*/*E* ≃ 10^−6^) [Fig. 3 ▸(*b*)]. Figure 3 ▸(*c*) shows a test result of scanning the self-seeded X-ray energy with an energy step of 1 eV while scanning the Si(111) DCM. The elastic scattering signals diffracted by the Si(660) analyzer crystals on the von Hamos spectrometer were equally spaced apart for different incident photon energies on the 2D detector, with the FWHM of the fitted Gaussian profile being ∼0.93 eV. The deviation of this bandwidth from 0.54 eV after deconvoluting the spectrometer energy resolution Δ*E*_VHS_ = 0.59 eV might be caused by the XFEL beam pointing jitter or uneven spatial distribution of the photon energies in each X-ray pulse (Kim *et al.*, 2022 ▸).

## 5. Ir *L*β_2_ RXES across the *L*_3_-absorption edge

The RXES is an experimental technique to measure the emitted X-ray spectrum from a sample while scanning the incident photon energy across an element-specific X-ray absorption edge (Kotani & Shin, 2001 ▸; Glatzel & Bergmann, 2005 ▸; Ament *et al.*, 2011 ▸; Castillo *et al.*, 2021 ▸). This technique involves a second-order optical process that includes X-ray absorption and fluorescence (Hayashi *et al.*, 2004 ▸; Kotani *et al.*, 2012 ▸). When an incident X-ray with a specific bandwidth interacts with the sample, it excites a core electron into an empty valence orbital or to the continuum (photoelectron state), creating intermediate states with a core-hole. These intermediate states rapidly decay through X-ray emission, which is measured using spectrometers equipped with analyzer crystals (Stojanoff *et al.*, 1992 ▸). The final states of the RXES process correspond to electron–hole pairs whose energies are transferred from the incident X-ray photon (Ament *et al.*, 2011 ▸). In the RXES experiment, the electronic structure of the sample is investigated by examining the intensity profile at a fixed fluorescence energy as a function of incident photon energies, and vice versa (Walroth *et al.*, 2016 ▸). For example, in the case of IrO_2_, incident X-rays with an energy around the Ir *L*_3_-absorption edge (∼11.22 keV) induce an electric dipole transition (2*p*_3/2_ → 5*d*) in Ir^4+^ ions (Horsley, 1982 ▸; Ping *et al.*, 2015 ▸). Subsequently, the 2*p*_3/2_ core-hole is radiatively annihilated by one of the outer-shell electrons following the selection rules. The emitted X-ray spectra are recorded using a crystal spectrometer like the von Hamos, and the resulting 2D intensity map in the incident versus fluorescence energy axis is interpreted by slicing it along one of the axes at a fixed value of the other axis (Rovezzi & Glatzel, 2014 ▸). The RXES technique allows access to the final states with an electron–hole pair in two different *d* orbitals, *e.g.*, a 5*d* excited electron and a 4*d* core-hole after the Ir *L*β_2_ (4*d* → 2*p*_3/2_) fluorescence, which are otherwise dipole-forbidden (Kalinko *et al.*, 2020 ▸).

**Figure 4:**
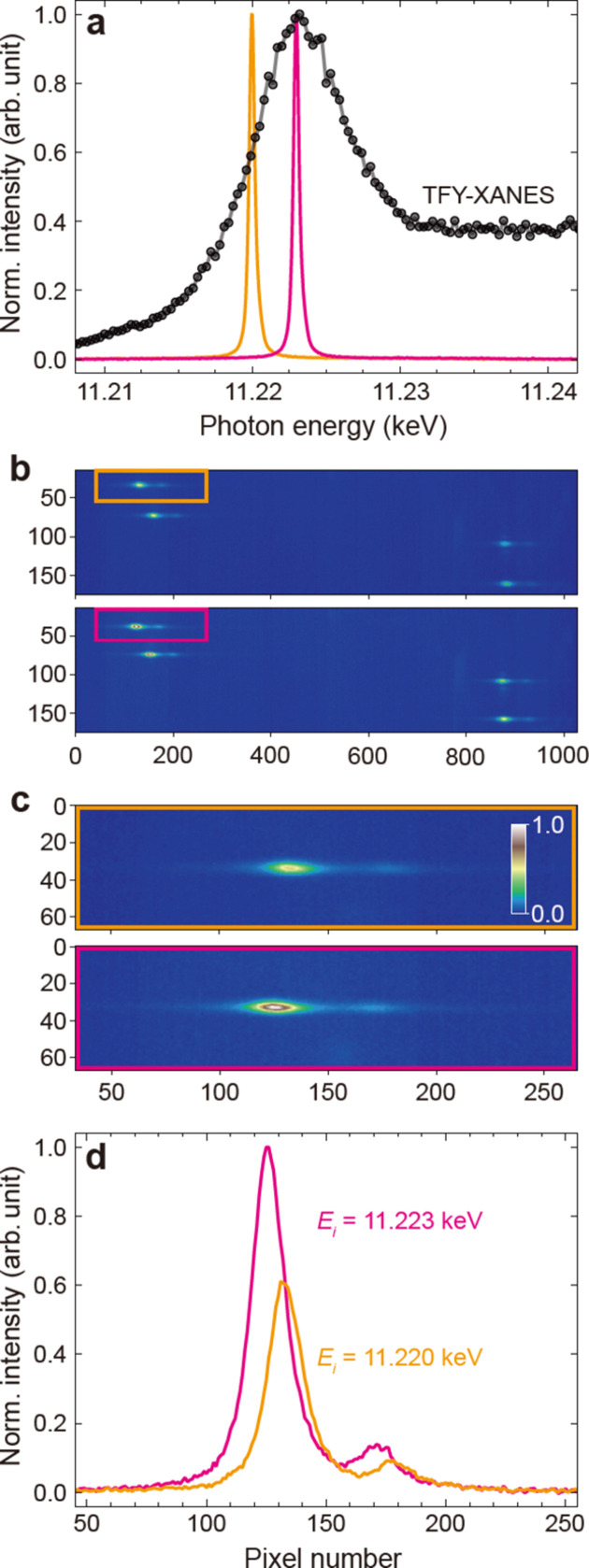
(*a*) TFY-XANES spectrum of IrO_2_ powder measured by scanning the incident photon energy with an energy step of 0.3 eV using the photon energy control system. The self-seeded X-ray spectra at 11.220 keV (orange) and 11.223 keV (pink) are plotted together. (*b*) Ir *L*β_2_ fluorescence signals of IrO_2_ diffracted by four von Hamos analyzer crystals, recorded simultaneously on the 2D detector. The upper (*E*_i_ = 11.220 keV) and lower (*E*_i_ = 11.223 keV) images are averaged over 1800 shots. (*c*) Magnified images of the orange and pink boxes in (*b*) from the same Si(660) crystal. (*d*) Ir *L*β_2_ X-ray emission spectra obtained by projecting the images in (*c*) onto the horizontal line.

The Ir *L*β_2_ RXES (or 2*p*_3/2_4*d* RIXS) experiment was conducted across the Ir *L*_3_-edge of IrO_2_ using the von Hamos spectrometer and the photon energy control system at the PAL-XFEL. IrO_2_ was chosen as a model system due to its significance as an oxygen evolution reaction catalyst with a 5*d* element (Ping *et al.*, 2015 ▸). The working principle of the photon energy control system was first verified by measuring a XANES spectrum in total fluorescence yield (TFY). Figure 4 ▸(*a*) shows the TFY-XANES spectrum of IrO_2_ powder with an energy step of 0.3 eV (black dots), overlaid with the self-seeded X-ray spectrum at 11.220 keV (orange) and 11.223 keV (pink). The photon energy axis was calibrated to literature data (Kalinko *et al.*, 2020 ▸).

Figure 4 ▸(*b*) shows Ir *L*β_2_ fluorescence signals on the 2D detector at incident photon energies of 11.220 keV (upper image) and 11.223 keV (lower image). The signals from Si(660) crystals are observed in the upper left, while those from Si(555) crystals are observed in the lower right. We note that Si(660) shows approximately twice as intense Bragg diffraction as Si(555) crystals. At 11.223 keV, a 2*p*_3/2_ core electron is excited into one of the unoccupied *e*_g_ antibonding orbitals of the Ir^4+^ ion, while at 11.220 keV it is excited into the singly occupied *t*_2g_ orbital (Horsley, 1982 ▸; Panda *et al.*, 2014 ▸; Ping *et al.*, 2015 ▸). Figure 4 ▸(*c*) provides magnified images of the orange and pink boxes in Fig. 4 ▸(*b*) for better comparison. The Ir *L*β_2_ X-ray emission spectrum diffracted by the same analyzer crystal varies with the incident photon energy, as shown in Fig. 4 ▸(*d*) by projecting the images onto the horizontal line. Two distinct emission lines ∼45 pixels apart decrease in intensity proportionally to the X-ray absorption and shift to lower energy as the incident photon energy changes from 11.223 to 11.220 keV. The horizontal axis in Fig. 4 ▸(*b*) was calibrated to the X-ray fluorescence energy for each analyzer signal, in accordance with the literature data (Kalinko *et al.*, 2020 ▸).

**Figure 5:**
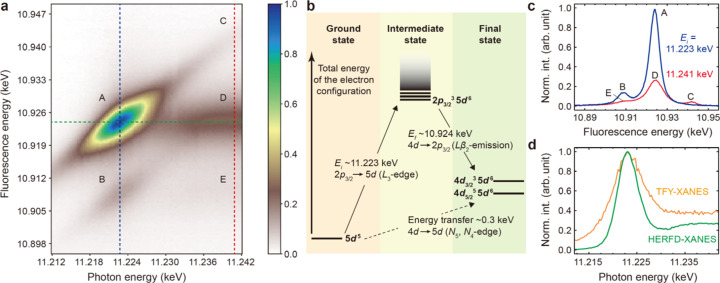
(*a*) Ir *L*β_2_ RXES map of IrO_2_ powder measured with four von Hamos analyzer crystals by scanning the incident photon energy of the self-seeded beam across the Ir *L*_3_-edge. Five spectral features are marked as A–E. The blue and red dashed lines are the Ir *L*β_2_ X-ray emission spectrum at *E*_i_ = 11.223 and 11.241 keV, respectively. The HERFD-XANES spectrum is obtained along the green dashed line at the fluorescence energy of 10.924 keV. (*b*) Energy-level diagram illustrating the Ir *L*β_2_ RXES (or 2*p*_3/2_4*d* RIXS) process in IrO_2_. The vertical axis represents the total energy within an Ir^4+^ ion. The final states with orbital occupations of 4*d* ^9^5*d* ^6^ are dipole-forbidden at the Ir *N*_5_- and *N*_4_-absorption edges. (*c*) Two representative Ir *L*β_2_ X-ray emission spectra from the RXES map at *E*_i_ = 11.223 and 11.241 keV. The features A–E are denoted. (*d*) The Ir *L*_3_-edge HERFD-XANES spectrum at the fluorescence energy 10.924 keV, which is plotted together with the TFY-XANES spectrum in Fig. 4 ▸(*a*) for comparison.

The incident-energy-dependent Ir *L*β_2_ X-ray emission lines of IrO_2_ were further measured across the Ir *L*_3_-edge by scanning the self-seeded X-ray energy from 11.212 to 11.242 keV with an energy step of 0.3 eV. The emission spectra from all four analyzer crystals at each incident energy were integrated, after aligning them to the respective X-ray fluorescence energy axis. The collected spectra normalized by their incident photon flux (= QBPM signal) are displayed as a 2D map in Fig. 5 ▸(*a*) with the maximum peak intensity set to 1. This mapping took an hour using the self-seeded X-ray pulses at a repetition rate of 60 Hz.

We note five spectral features A–E in the Ir *L*β_2_ RXES (or 2*p*_3/2_4*d* RIXS) map. In the second-order optical process of the RXES, as depicted by the energy level diagram in Fig. 5 ▸(*b*), the incident energy determines the intermediate state upon X-ray absorption, while the fluorescence energy indicates the final state (Kotani *et al.*, 2012 ▸). The spectral features A and B have their local maximum intensity at *E*_i_ = 11.223 keV (blue dashed line) and share the same intermediate state with a 5*d* excited electron and a 2*p*_3/2_ core-hole. However, their final states are different by 16 eV. The energies transferred from the incident X-ray photon in these two final states are 299 eV (A) and 315 eV (B), which are close to the Ir *N*_5_- and *N*_4_-absorption edges, respectively (Chantler, 1995 ▸). Thus, the feature A (B) involves a 4*d*_5/2_ (4*d*_3/2_) core electron refilling the 2*p*_3/2_ core-hole radiatively. These two discrete final states show a Raman shift along a diagonal direction in Fig. 5 ▸(*a*) due to the 2*p*_3/2_ core-hole lifetime broadening (Glatzel *et al.*, 2009 ▸; Gretarsson *et al.*, 2011 ▸). The X-ray absorption to an empty 5*d* orbital also occurs at lower and higher incident photon energy within the 2*p*_3/2_ core-hole lifetime broadening, followed by the Ir *L*β_2_ resonant X-ray emission at lower and higher fluorescence energy, respectively (Krause & Oliver, 1979 ▸; Hämäläinen *et al.*, 1991 ▸). The Raman feature from A is still weakly present in the feature C at *E*_i_ = 11.241 keV (red dashed line). At this photon energy, a 2*p*_3/2_ core electron is excited to the continuum (photoelectron state), and a 4*d*_5/2_ (D) or a 4*d*_3/2_ (E) core electron refills the 2*p*_3/2_ core-hole.

The features A–E are summarized in Fig. 5 ▸(*c*) by two representative Ir *L*β_2_ X-ray emission spectra at *E*_i_ = 11.223 and 11.241 keV [the blue and red dashed lines in Fig. 5 ▸(*a*), respectively]. Each emission line in both spectra is broadened predominantly by the 4*d* core-hole lifetime (Keski-Rahkonen & Krause, 1974 ▸; Krause & Oliver, 1979 ▸; Glatzel *et al.*, 2009 ▸). Figure 5 ▸(*d*) shows the Ir *L*_3_-edge HERFD-XANES spectrum corresponding to an intensity profile at the fluorescence energy of 10.924 keV in Fig. 5 ▸(*a*) (the green dashed line). The TFY-XANES spectrum in Fig. 4 ▸(*a*) is also plotted for comparison (orange line). The HERFD-XANES spectrum exhibits a narrower spectral bandwidth compared with the TFY-XANES spectrum, as indicated by a shallow dip visible at *E*_i_ = 11.230 keV (Hämäläinen *et al.*, 1991 ▸; Glatzel *et al.*, 2009 ▸; Kotani *et al.*, 2012 ▸).

**Figure 6:**
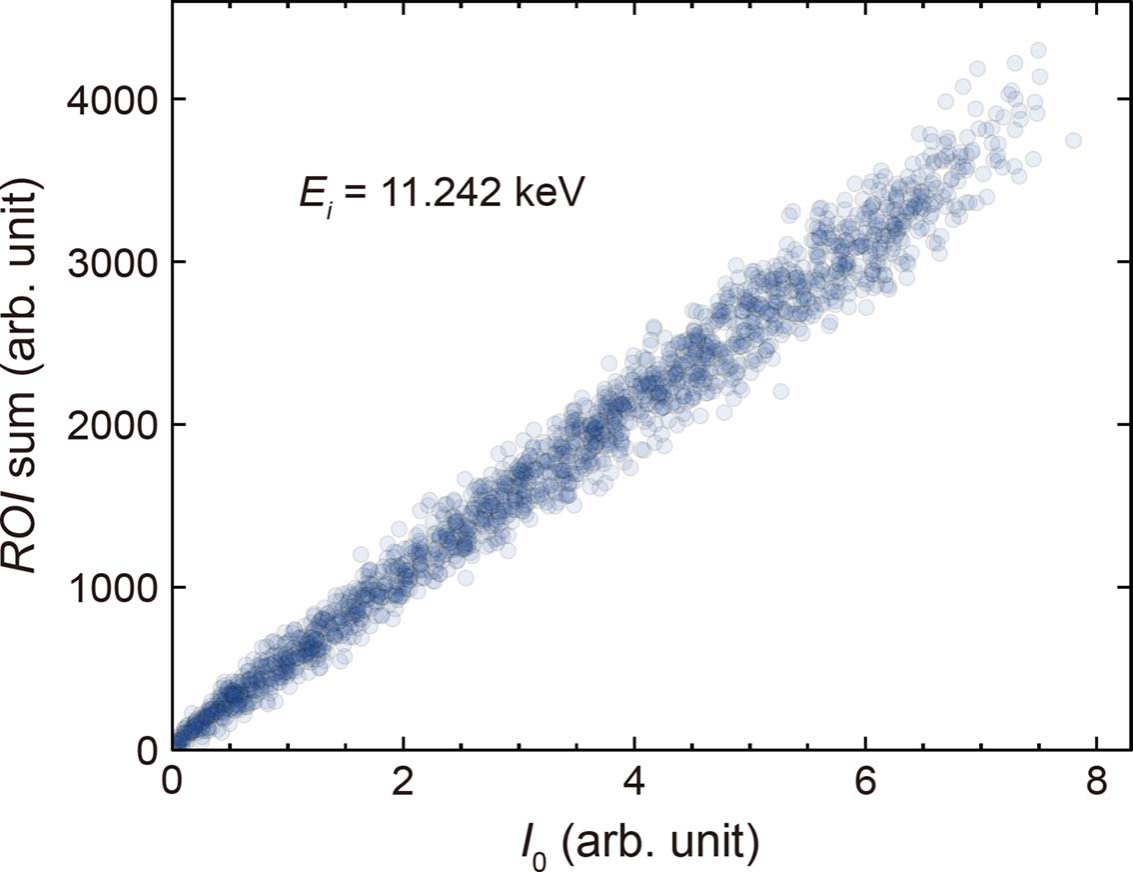
Single-shot intensity correlation between the QBPM signal (*I*_0_) and the sum of counts in a ROI on the JUNGFRAU detector over 18000 shots at *E*_i_ = 11.242 keV. The ROI is set as a box of 11 × 23 pixels centered on the pixel with the maximum Ir *L*β_2_ fluorescence intensity from one of the Si(660) analyzer crystals. The QBPM signal is normalized by the average photon flux of the SASE beam, as in Fig. 2 ▸(*c*). The average value of the slope is 515.82 with standard deviation of 1.66.

Figure 6 ▸ presents a single-shot intensity correlation between the QBPM signal and the sum of counts in a region of interest (ROI) on the JUNGFRAU detector over 18000 shots at *E*_i_ = 11.242 keV. The ROI was set as a 11 × 23 pixel box centered on the pixel with the maximum Ir *L*β_2_ fluorescence intensity from one of the Si(660) analyzer crystals. The QBPM signal serves to normalize the fluctuation of the incident photon flux based on its linear response to the emitted X-ray intensity. The average value of the slope is 515.82 with its standard deviation of 1.66. This observation implies that a transient change over 1% in a time-resolved XES experiment is discernible at the hard X-ray beamline of PAL-XFEL.

## 6. Conclusions and outlook

This work presents the first commissioning results of an Ir *L*β_2_ (4*d* → 2*p*_3/2_) RXES experiment across the Ir *L*_3_-absorption edge (2*p*_3/2_ → 5*d*) of IrO_2_, using self-seeded hard X-ray pulses at the PAL-XFEL. A point-to-line energy-dispersive von Hamos spectrometer equipped with four cylindrically bent crystals was employed at the XSS hutch to diffract the X-ray emission signals. The photon energy control system for the self-seeded hard X-ray beam was utilized to scan the incident photon energy by coordinating the electron bunch energy and the diamond [100] crystal angle together with the Si(111) DCM. The scanning scheme from 11.212 to 11.242 keV led to a successful mapping of the Ir *L*β_2_ resonant X-ray emission lines within an hour at a repetition pulse rate of 60 Hz. The Ir *L*β_2_ X-ray emission spectrum at the maximum X-ray absorption reveals two spectral features involving the 4*d*_5/2_ and 4*d*_3/2_ core-hole final states, which exhibit a Raman shift due to the 2*p*_3/2_ core-hole lifetime broadening. The experiment also deduced the HERFD-XANES spectrum across the Ir *L*_3_-edge. This spectrum demonstrates a narrower absorption feature than the TFY-XANES spectrum. Furthermore, a linear intensity correlation was established between the incident photon flux and the emitted X-ray signal. This correlation will allow one to distinguish a transient change over 1% in a time-resolved XES experiment.
